# On the Etiology of Epidemic Cholera

**Published:** 1833-09

**Authors:** Alexander Turnbull Christie

**Affiliations:** the Honourable East-India Company's Service; Member of the Royal Asiatic Society of Great Britain and Ireland, of the Wernerian and Royal Medical Societies of Edinburgh, &c.


### THE EPIDEMIC CHOLERA.

*On the Etiology of Epidemic Cholera.* By Alexander Turnbull Christie, M.D., of the Honourable East-India Company's Service; Member of the Royal Asiatic Society of Great Britain and Ireland, of the Wernerian and Royal Medical Societies of Edinburgh, &c.

(Continued from page 100.)

I am aware it may be objected to the view I have taken of the subject, that in many cases of cholera there has been neither vomiting nor purging. I will shew, however, that the catarrhal affection of the mucous membrane is not only present in such cases, but is even sometimes greater than in those cases in which vomiting and purging are most urgent. I have opened the bodies of patients in whom the symptom of vomiting had been entirely wanting, and found the stomach filled with serous fluid, and its inner surface lined with a white coagulum.

CASE. Shaik Ebram, a convict, aged thirty-six, when at work in the open air, on the 9th May, was attacked with purging at two P.M. He was brought into hospital at half-past four o'clock; up to which time he had been purged only four times, and had not vomited once. He was weak. He exhibited no signs of pain or uneasiness; and, although unwilling to be roused, was perfectly sensible. No pulse at his wrist; features collapsed, and skin cool. Spasms did not occur throughout the case, except a slight twitch, of short duration, in the muscles of the loins.

It is unnecessary to give a minute detail of the treatment. Suffice it to say, that I attempted to bleed him from the arm and temporal artery, but could only procure an ounce of dark-coloured blood from the former. He took two scruple doses of calomel, some tincture of cardamoms, cajeput oil, and camphor mixture. Boiling water was applied to the belly, and sinapisms to the legs and feet; but nothing was of the slightest avail. He died at six o'clock.

His body was examined the same evening, a few hours after his death, and presented the following appearances: Considerable congestion of the veins of the stomach and mesentery. The stomach contained a very large quantity of serous fluid, and a small quantity of food, and its mucous coat was white, lined with coagulated fibrin,* and exhibited a little redness near the pylorus. The intestines were in many places contracted: they contained a large quantity of white and transparent serous fluids, and their mucous membrane was lined with a white coagulated fibrin throughout its whole extent. The liver was healthy. The gallbladder contained healthy bile. Considerable congestion of the meningeal veins. No inflammation in any of the contents of the cranium. The tuber annulare, and upper part of the spinal marrow, were healthy.

About the same time another convict died of cholera, who had no vomiting, and in his stomach I found a larger quan-

* The secretions in this case were examined chemically, and afforded the results already stated.

tity of the coagulated secretion than I had ever observed in any other case. Thus we see that, although there be no vomiting, there is nevertheless a severe catarrh of the gastric mucous membrane. The same observation, I am convinced, will hold good respecting the state of the enteric mucous membrane as connected with purging. Mr. Scot, at page 13 of his Report on the Epidemic Cholera, says, "In cases where little or no purging has taken place during life, the intestines have yet been found, after death, to be filled with the congee-like matter, as if they wanted energy to throw it off, or as if a stricture had been formed on the lower portion of the gut." Indeed, the case of Shaik Ebram also, to a certain extent, proves the same thing; for in it the purging was very trifling, and the quantity of diseased secretions in the intestines was very great. But this case is still further useful, by shewing what an immense quantity of the diseased secretion may be thrown out by the mucous membranes in a very short time; and also that these diseased secretions are sometimes the only morbid appearance of any consequence in cholera.

Let us now inquire how the catarrhal affection, which we have shewn to be invariably present in cholera, produces the other morbid changes, and the symptoms of the disease: and first, respecting the changes in the circulation; the congestion in the veins of the viscera has been already explained. This we have stated to be of invariable occurrence in severe cases, which we might have been led to expect, from the discussions we have entered into upon the etiology of mucous membranes. The greater the discharge from the alimentary canal, the stronger and more abundant will be the current of blood directed towards it, to supply the waste; and the vessels at the surface being thus drained of their contents, all those appearances of collapse, shrinking, with loss of pulse, which have been described among the symptoms, must necessarily take place. But the danger of every disease depends in a great measure upon the rapidity of the invasion; and this is particularly the case in cholera; for the rapidity of the determination towards the viscera being in proportion to that of the catarrhal affection, the former may occasion such a shock to the vital organs as to prove soon fatal. When, on the other hand, the disease approaches gradually, commencing with nausea, or an insidious diarrhœa, the determination of blood towards the viscera takes place slowly, and the circulation is kept up tolerably well towards the surface for some time. Lastly, when the invasion is ushered in with inflammation, the pulse is at first strong and full, and symptoms of congestion do not occur until the catarrhal affection is established.

Having accounted for the congestion in the abdominal viscera, it may now be asked, what occasions the venous congestion in the thorax and encephalon? I will answer this by putting another question: what occasions a similar congestion in animals which have been bled to death? It would appear that the abstraction of a very large quantity of the circulating fluid tends to cause the remainder to accumulate in the internal parts: how it does so, is another question; but, if the fact be allowed, it exactly accords with my views of cholera; for I have shewn that the quantity of the blood is much diminished by the drain from the mucous membranes. I imagine the congestion in the encephalon generally does not take place until a very short time before death; for we can scarcely suppose that the mental faculties and the senses would remain so unimpaired as they frequently do, until within a few minutes of the fatal termination, if the brain were loaded with blood.

Next to congestion, the most important morbid appearance is the altered condition of the blood; which also receives a clear and satisfactory explanation from the diseased secretion of the mucous membrane. It would have been wonderful, had the blood retained its healthy appearance after being deprived of so large a quantity of serum and fibrin; and are not the diseased appearances observed in it exactly what we might have expected, *a priori*, from the abstraction of so much of these substances? It must necessarily become thicker when deprived of an inordinate quantity of serum, and darker, from none of its colouring matter being discharged, the fibrin which is secreted being always white. It has been sometimes said to have been thin and watery; but this is of rare occurrence, and may have been owing to the cholera secretion containing a larger quantity of fibrin than usual.

The explanation of the diminished secretion of urine and saliva is evident. It must be referred to the small quantity, and perhaps also to the altered condition, of the blood which is sent to the kidneys and salivary glands. All parts of the body are robbed of their due proportion of the circulating fluid to supply the expenditure of the mucous membranes; and the little they do receive is vitiated, and therefore ill suited to administer to their healthy functions.

But we have still to account for an important symptom, which often forms a prominent feature in the disease; I allude to the copious cold sweat. The skin is properly considered as a continuation of the mucous membranes, which it resembles in its structure and functions. The different parts of the mucous system, including the skin, are linked together by sympathies; one of the most conspicuous of which is that which exists between the gastro-enteric membrane and the periphery. When the action of the former is increased by an emetic, by a purgative, or even by warm fluids, the latter sympathises, and the cuticular secretion is increased. This is a wise provision of nature, whereby the tendency to congestion, from the determination of blood which takes place towards the mucous membranes, is lessened, and a copious perspiration is in this way often a most salutary operation, whether excited by art or by nature. But, in cases of epidemic cholera, the perspiration, however copious, is seldom capable of counteracting the congestion caused by the overwhelming catarrh of the mucous membranes; and the cold clammy sweat has accordingly been sometimes rather figuratively, but perhaps not inaccurately described as an ineffectual effort of struggling nature to restore the circulation towards the surface. It is indeed remarkable that there is sometimes a copious perspiration when the pulse at the wrist is extremely small, or nearly imperceptible. This has generally been considered as indicative of extreme debility of the perspiratory vessels. On the other hand, I am inclined to think that these vessels have their action very much increased; for, were the reverse the case, how does it happen that, when only a small quantity of blood flows sluggishly, or in drops, from a large orifice made in a vein, or even when no blood can be procured, the cuticular secretion forces its way through the minute pores of the skin? When the arteries do not contain a sufficient quantity of blood to enable them to continue their own action, the only way in which perspiration can be thrown out is evidently by an increased action of the perspiratory vessels. But perspiration is by no means an invariable symptom of cholera; for in some cases there is only a slight moisture about the face and hands, and in others the whole skin is perfectly dry.

It is an interesting fact, that a similar phenomenon to the above takes place in animals which have bled to death. Sir Charles Bell says: "If a glass be attached to the artery of a horse, the column of blood will suddenly rise in the tube, marking the force of circulation. If the animal be bled from another vessel, the blood will descend in the column as the force of the circulation is enfeebled. When the blood is very low, and the animal dying, the surface will break out profusely in perspiration. It must be concluded, that the force of the internal impulse is not the cause of perspiration. Again, the most persevering efforts in injecting the vessels will never bring the water through the pores of the skin; and if a dead body be carried near the fire, the heat will not bring out one drop of fluid from the pores of the cuticle."* Sir C. Bell therefore concludes that the perspiration is produced solely by the vital action of the perspiratory capillary vessels.

Since the epidemic cholera is a disease of the mucous system generally, and since the skin is considered to belong to this system, the increased discharge from the latter may be considered not to arise from sympathy, as already conjectured, but rather as a simple extension to the periphery of that morbid action of the internal membranes which constitutes the disease. But the laws of sympathy are so obscure, and so little understood, that both of these opinions may, for all we know, amount nearly to the same thing, and either may be adopted consistently with our theory. It will be necessary here to take notice of the following argument, which has been urged against these views. It has been said, "it seems but reasonable to expect that, if a disease similar to that which produces such an afflux to the interior existed simultaneously on the surface, there would be a division of the blood between the centre and the periphery, and, instead of smallness or imperceptibility of the radial pulse, we should expect to find it tolerably forcible."† The answer is very simple and obvious: such a determination is generally prevented by the great intensity of the disorder in the internal membranes, which form a surface more extensive than the whole of the skin, and throw off a quantity of fluid far superior to that of the most copious sweats. Nevertheless, the blood is sometimes directed towards the surface; but then what takes place? There cannot be both a determination of blood towards the interior and towards the periphery at one and the same time: the balance of the circulation is therefore restored, and the disease ceases.

A very distressing symptom, which occasionally occurs in cholera, is difficulty of breathing. It is plainly to be attributed to the obstruction of the minute bronchia by their diseased secretion: it cannot be referred to spasm, for it often occurs in cases in which spasms are entirely wanting. The stethoscope might afford us some assistance in the diagnosis of this part of the disease.

* An Essay on the Forces which circulate the Blood, by CHARLES BELL. London, 1819.

† Medico-Chirurgical Review, No. 19, December 1828, p. 126.

Coldness of the skin, which is one of the most characteristic symptoms in the advanced stage of the disease, is clearly owing to two causes: 1st, to the diminution in the quantity of blood sent towards the surface; 2dly, to the altered condition of the blood.

Debility of the voluntary muscles is a common symptom of cholera; but in many cases it is by no means so great as we might be led to expect from the very extensive diseased condition of the mucous membrane. Sometimes, when the pulse is scarcely perceptible at the wrist, the patient is still able to walk; and, long after the pulse has ceased at the wrist, (although perhaps not able to move about,) he is often sufficiently strong to sit up, to use his arms and hands, to speak, &c.

When we consider the great diminution of muscular power that is sometimes occasioned by simple vomiting, by a diarrhœa, or by a large dose of tartrate of antimony, we cannot be surprised at the great debility that often occurs in cholera; and the only cause of astonishment is, that this debility is not invariable. This, however, in concurrence with the perfect state of sensation, clearly shews that the debility of the muscles is not owing to diminished energy of the nervous system. A person is attacked with cholera; the blood gradually leaves the surface, which, as might be expected, occasions more or less muscular debility; his mental faculties, however, remain undisturbed;* all the external senses continue perfect; and, in fact, almost all the functions of the nervous system are for a long time unimpaired, until, near the fatal termination, they become affected by the great derangement of the circulation.

In some cases of cholera, anxiety, and an overpowering sensation at the præcordium, generally denominated sinking, are very distressing symptoms. The latter is, most probably, only a variety of the former, from which it appears principally to differ in being more severe. Both may be attributed either to the disordered state of the stomach or of the lungs; sometimes, perhaps, they are referrible to the one, sometimes to the other.

Spasms have been by no means of invariable occurrence in the cholera of India, and are clearly referrible to irritation in the gastro-enteric mucous membrane, whether this proceeds from inflammation, or a sudden determination of blood towards the parts, in consequence of the catarrh. They are

* Vide Annesley's Sketches of the Diseases of India, p. 21; also Anomah's case, p. 58, and the cases in the Appendix.

almost always present in cholera morbus, and in mixed cases, and are much more frequent in European than in native patients, owing to the greater irritability and tendency to inflammation in the former.

When we consider what violent spasms arise from comparatively trifling irritation of the primæ viæ, we cannot be surprised at their frequent occurrence in cholera. The absence of spasms in those cases in which there is only catarrh of the mucous membranes, without inflammation, is quite consistent with what we know of the nature of catarrh; for it never directly produces any sensible effect on the nervous system.

If, in addition to the catarrhal affection, inflammation be present in the mucous membranes, the symptoms of the disease will of course be more or less modified. If the inflammation be not very violent or extensive, it will probably be indicated only by local pain or burning, or perhaps merely by pain on pressure. If it be great, it will excite the action of the general circulation.

Frequently pain in the abdomen does not occur until a late stage of the disease, and in these cases is probably owing to inflammation. It has been observed by several medical men that calomel is often found adhering in patches to the inner surface of the stomach of those who had died of cholera, and in these places only was there inflammation. In several cases I have found appearances of inflammation in the pyloric extremity of the stomach, where the stimulating medicines given for the cure of the disease were collected. It would appear, therefore, that inflammation is sometimes occasioned solely by the violent remedies used for the cure of the disease.

It is a curious feature of many cases of cholera, that there is great thirst, while the mouth at the same time is perfectly moist. It is however by no means an invariable symptom; and I am inclined to think that it is sometimes occasioned by the stimulating medicines given to the patient, and sometimes to inflammation being conjoined to the catarrhal affection of the stomach; for it is found to be most urgent in cases where there is great pain at the præcordium.

Cholera morbus is accompanied with increased action of the circulation, hot dry skin, &c. In it, therefore, there ought to be (consistently with our pathological views,) acute inflammation of the mucous membrane of the primæ viæ. The few authors who have taken notice of the post-mortem appearances in this disease state that marks of inflammation were found in the mucous membrane of the primæ viæ; and the symptoms of inflammation of this membrane are so well marked during life, that we scarcely require more positive proof of its presence. To what else can we refer the burning at the epigastrium, pain of abdomen, especially on pressure, heat of skin, and quick, hard pulse? Moreover, the vomiting and purging in cholera morbus are very different in their nature from those which occur in the catarrhal form of the disease; for they never contain the sero-fibrinous secretions which characterize the latter, but consist principally of bile.

That cholera morbus consists of an inflammation of the gastro-enteric mucous membrane, may perhaps appear questionable, from the circumstance of its generally running its course very rapidly. But it appears to me that this is exactly what we might be led to expect from the nature of the disease; for, if the mucous membrane of the primæ viæ be suddenly affected with inflammation, a rapid determination of blood takes place towards it; and, since it possesses an excretory apparatus, an evacuation will probably be thrown out from its extensive surface, whereby the inflammation will be quickly subdued, and the disease removed; or, if the evacuation be inordinate, it may degenerate into catarrhal cholera, and thus soon prove fatal.

All the other symptoms which occasionally occur in cholera are of minor importance, and do not require to be taken into account in a general view of the pathology of the disease. It is necessary however to advert, in a general manner, to those affections which arise from accumulation of blood in the head, such as apparent coma, tennitus aurium, deafness, &c.; for to this accumulation some have referred the proximate cause of the disease. But, far from its being the proximate cause, it is by no means a general occurrence; and in no case does it occur until the latter stages of the disease, and until after all the other symptoms have been developed.

If the preceding pathological views be found to be correct, it will follow that there are two essentially different kinds of cholera: one, the disease usually denominated Cholera Morbus, or Cholera Biliosa, consisting of an inflammation of the gastro-enteric mucous membrane; the other, the Indian Cholera, or Cholera Asphyxia of Scot, consisting of a violent catarrh of the mucous membranes generally; and, further, that cases sometimes occur of a mixed nature, from catarrh and inflammation being present in the mucous membranes at the same time. Should these views be adopted, the correct designations of the two principal species of cholera would be Cholera Pyretica and Cholera Catarrhalis; the former being the cholera morbus, the latter the epidemic cholera, (Cholera asphyxia of Scot,) and Cholera spasmodica of some other authors.*

As it is the opinion of some that, in my former work on the Pathology of Cholera, I have allowed to the nervous system too little participation in the phenomena of the disease, I beg leave to state here what are my exact views on this subject. I conceive that every part of the animal frame ought to be considered as a compound body, made up of a certain number of essential elements, such as nerves, blood-vessels, capillaries, &c., all of which act and react on each other, and no one of which can be affected in any way without influencing the rest. Now, in a great many diseases, (although perhaps not in all,) I would be inclined to refer the morbid condition to the compound body of nerve, blood-vessel, and capillary, of which the affected part consists, not to any one of these elements exclusively; just as we refer the phenomena of galvanism to the compound arrangement of two metals and an interposed fluid, and not to any of them taken alone. When I say, therefore, that the cholera is a disease of the excretory apparatus of a mucous membrane, I mean that it is a disease of a certain complicated arrangement of nerves and vessels, which requires the concurrence of all its elementary parts for the proper performance of its functions. These act together to produce certain results, and the derangement or abstraction of any one of them would certainly derange the whole. Let us suppose that the usual powerful and striking effects of our galvanic battery should become enfeebled, we should probably find, upon examination, that this arose from oxydation of some of its plates; but, not content with this discovery, we might push our inquiry a little further, and find that the oxydation itself has been caused by the acid of the interposed fluid. So, in cholera, we have discovered that the disease depends upon an affection of the mucous membranes; and further investigations *may* enable us to make another step, and to demonstrate this affection to be caused, in its turn, by some

* Consistently with these principles, cholera may perhaps be considered objectionable as a generic term. But, if (instead of deriving it from χολη, *bilis*,) we adopt the derivation of Trallian,[a] Castellus,[b] and others, viz. of χολας, *intestinum*, the term will then bear the accurate signification of intestinal flux; and to which the above terms, Pyretica and Catarrhalis, will serve as appropriate specific adjuncts.

[a] Vide Good's Study of Medicine, second edition, vol. i. p. 260.

[b] Nomen habet non tam αχολη, quam αχολας, i.e. intestinum, per quod materia ex ventre excerrutur.—Bertholomæi Castelli, Lexicon Medicum. Lipsiæ, 1713.

peculiar state of the nerves. Should this be clearly made out, I should be the first to hail with pleasure so important an addition to our knowledge of pathology; but I must maintain, that at present we have no proof of any affection of the nervous system forming the first link of the disease. This is an inquiry, however, that is highly deserving of being prosecuted; for there are many familiar facts which shew that the nervous system exerts the most important influence over the actions of the capillary vessels, and especially of those which produce the secretions. I need only mention the action of blushing from shame, the secretion of tears from grief, the secretion from the stomach by disgust. The valuable observations of that excellent philosopher, Sir Charles Bell, on the nervous system, and the late brilliant discoveries in animal electricity and electro-magnetism, would incline us to believe that we are on the very verge of some great discovery on the philosophy of life, which will probably enable us to ascertain the exact influence (no doubt very great,) which the nerves have in the production of the various functions of the animal frame. Our knowledge of the pathology of cholera may be compared to that which the chemists possess in regard to some substances which are *suspected* to be compound, but which have not yet been analysed; and, notwithstanding that their compound nature is admitted to be probable, yet chemists continue to treat them, and to reason upon them, as if they were elementary. We have analysed the cholera to a certain extent, and we may in time gain possession of a formula by which we shall be able to reduce it to still more simple elements: meanwhile, do not let us wander into the regions of conjecture, indulging in wild theories, which might turn us away from the sober but sure path of inductive reasoning and experiment, which alone lead to truth.

### *Causes of Cholera.*

Having pointed out the nature of the disease, and demonstrated, I hope satisfactorily, that it has its seat in the mucous membranes, I will now proceed to inquire into its external causes, and the mode in which it is propagated.

Although epidemic visitations had not been of frequent occurrence before the year 1817, sporadical cases had often been met with in India, and had been almost invariably attributed to atmospherical vicissitudes. The disease is generally most prevalent during changeable weather, and ceases when the weather becomes serene and temperate. It usually makes its attack during the night, or early in the morning, especially if the individual has been fatigued during the day; and it is notorious that it is most prevalent among troops when marching, for they then undergo more fatigue, and are more exposed to the cold night-air than usual. In these respects, then, it strongly resembles catarrh, diarrhœa, and dysentery. But it is also excited by a different set of causes; viz. by such as, instead of acting on the skin, are applied directly to the mucous membrane of the primæ viæ. Thus, it has often arisen from a large dose of salts; unripe fruit has been known to cause it; and it has very often been induced by a large draught of cold water, buttermilk, &c. Its causes, then, may be said to be such as excite a catarrh in the mucous membranes, especially in those of the primæ viæ.

But, after the disease became fairly established as an epidemic, it broke through all its former laws, sparing neither sex, age, nor rank; prevailing at all seasons, and visiting all situations and all climates. We therefore now arrive at the important question, whether it is capable of being propagated by contagion or infection?* On a slight consideration of the subject, one might be inclined to believe the disease to be infectious or contagious; for, after its appearance in Bengal, in 1817, it gradually spread thence from place to place, till it reached the most distant parts of India, China, and all the islands of the Eastern Archipelago, Persia, and at length Russia; checked neither by climate, contrary winds, nor intervening seas. Upon examining the question a little more closely, however, we shall find that numerous facts and arguments are hostile to this belief; a few of which I will now proceed to examine.

When spreading across a tract of country, the disease has often been known to spare one or two towns, and to appear in the next, although all must have been equally exposed to the infection, had any existed. It has been known to rage among troops in a town, while the townspeople escaped; or to attack the townspeople, and to spare the soldiers. A troop of horse-artillery, of which I had charge at Kulladghee, in 1824, suffered severely for about a fortnight from the disease; while all the other corps at the station, although maintaining an uninterrupted intercourse with the artillery, were exempt. My dresser, who lived in a room attached to the hospital, caught the disease, and died; but not one of the other patients who lay in the same ward with those labouring

* These terms being variously employed by different authors, I find it necessary to state that, by a contagious disease, I mean one which is capable of being communicated by contact; by an infectious disease, one which can be conveyed from one person to another through the medium of the air.

under cholera were attacked. I have repeatedly remained for hours by the bedside of cholera patients, and never received the infection.

A regiment has been frequently known to march through a country, when suffering from cholera, without communicating it to the inhabitants; and, on the other hand, the disease has often prevailed in a country, without attacking the troops which passed through. It has very often happened that a single individual in a family has caught the disease, without communicating it to the rest. Of two corps on a march, and keeping up the most unreserved intercourse, one shall suffer from the disease, and the other escape. "Detachments of a regiment, arriving from a particular place, suffer severely; while the rest of the regiment, which has remained stationary, shall hardly furnish a single case, although the former may be living in the same barracks, and their sick in the same hospital."* Now, although facts like these, if taken singly, might not perhaps be thought conclusive, yet, when it is recollected that they have been of constant occurrence ever since the first appearance of the epidemic, we must, I think, consider them, when united, as affording strong evidence against the infectious or contagious nature of the malady.

It has been supposed by some medical men that the epidemic was introduced into the Mauritius by ships from India; but it is sufficient to state, in opposition to this, that the commission of medical officers appointed by the government of that island to investigate the nature of the distemper, gave it as their decided opinion that it was not contagious. Notwithstanding the strictest quarantine having been insisted on in the island of Bourbon, the disease appeared there in January 1820. The minds of the people being then filled with notions of contagion, supposed it to have been introduced by means of a boat which had communicated with a ship from the Mauritius; but this was merely a supposition, unsupported by proof. That it was introduced in a similar manner into Trincomalee, is also highly improbable; and it has been already mentioned that it had existed in Ceylon for more than a year previously to the arrival of the infected ship which was said to have imported it. I need only refer to the general history of the epidemic, as confirmatory of its non-contagious nature. Thus, in its progress through the Indian islands, it sometimes visited the most distant first; just as it had previously proceeded on the continent of India, where several towns on a river, or high road, were

* Madras Report.

passed over, and those at a distance beyond were first attacked. The progress of the epidemic through Persia, Syria, and Russia, abounds with examples of the same kind.

Amongst its first victims in Shirauz were the wife, mother, and child of the prince of Persia, who, confined to their haram, were little likely to be exposed to contagion or infection. Another case of a similar description, but still more remarkable, has been already mentioned; viz. that the city of Oudeypore had long continued free from the disease, but, while the inhabitants were congratulating themselves with the belief that they were beyond its western limit, they were surprised and terrified at the intelligence of their prince and his minister having been attacked by it in the very palace, while the city was still perfectly healthy. Such being the case, what would avail the most careful sanitary cordon, and the strictest quarantine?

The following paragraph is from the work of Mr. Annesley, whose extensive experience must entitle his opinion to the highest consideration.

"As the non-contagious nature of the disease is very generally admitted by the medical authorities of India, who have had sufficient experience of the disease, and as this property is generally believed in by the community at large, I should not have thought it necessary to advert to a contrary opinion, had not that opinion received the support of some distinguished medical authorities. I cannot, however, but think it unfortunate that the idea was ever suggested, because the dread of contagion may lead to serious consequences; inasmuch as it may withhold from the sick that assistance which they so much require. The strongest proof which I can adduce in opposition to it came fully under my own experience, which has not been inconsiderable, and was derived chiefly from what I observed in the general hospital at Madras, whilst it was under my charge. This hospital generally contained from 170 to 200 patients, natives and Europeans; the wards were open, and a free communication existed between them; and yet, although patients were daily brought into them, suffering under epidemic cholera,—although these patients were indifferently distributed throughout the hospital, and consequently not secluded from the rest of its inmates, no more than five or six persons, exclusive of the two already noticed, were seized with the disease, while patients in the hospital, during a period of five years; and certainly these cases could not be imputed in any degree to contagion. I can view them merely as cases of cholera occurring under circumstances of predisposition, during the prevalence of an epidemic cause, and as shewing even a much diminished ratio of attack to that observed where the disease prevailed."

Sir Gilbert Blane, in a letter to the court of directors of the East India Company, in 1825, advocates the theory of contagion; but, while we admit the great consideration that so high an authority is entitled to, we must keep in mind that he had never an opportunity of attentively watching the progress of the disease in the countries where it prevailed; and his single opinion is opposed to that of almost all the distinguished surgeons of India.

Monsieur Moreau de Jounes also, in a communication to the Institute of France, on the 22d of November, 1830, professes his belief in the contagious nature of the cholera, derived from the history of its progress through the countries it has already visited; and, consistently with this notion, he supposes it to have been introduced into Oremburg, in Russia, by the caravans which bring from Bokhara the merchandise of Thibet, Cabriel, and India. But Baron Humboldt, who was present at the meeting, stated, in opposition to M. Moreau, that the cholera had not made its appearance at Oremburg before his departure from that town, although the caravans had arrived nearly four months before; and that the plains of the Kirguises, which these caravans had traversed, had not been infected with the malady. It may be also mentioned, that the inhabitants did not suppose it to have been imported, but to have been produced by atmospherical influences.

I would observe, then, in conclusion, that the only plausible argument for supposing the disease to be contagious or infectious is, that it has spread from Bengal, as a centre, in a very gradual manner, to distant countries, and has prevailed in all climates and situations; but that in this respect it does not differ from other epidemics, some of which have had an equally wide diffusion, and which are always admitted to be non-contagious. Further, many facts already detailed respecting the progress of the disease are perfectly irreconcileable with the theory of contagion or infection; and those few which appear favourable to this doctrine are readily explained by the opposite. I have therefore no hesitation in giving it as my decided opinion, that cholera is neither contagious nor infectious, and consequently, that quarantine and sanatory cordons will prove completely inadequate to check its progress.

The disease has been supposed to arise from malaria; but this opinion will be found to be as untenable as that which supposes it to be contagious. Some sporadic cases have doubtless had the appearance of having been produced by malaria; but this has been by no means the case with the epidemic, the progress of which in India was often in direct opposition to the prevalent periodical winds. It often rioted in the low, confined, and crowded abodes of the poor; but they have escaped when it has penetrated into the spacious airy dwellings of the rich; and occasionally (although seldom) it has extended to lofty dry situations, while the low damp country around was spared. During the prevalence of the epidemic at Madras, "the labourers at certain public works, who were protected from the weather, who were well clothed and fed, and who had no unusual work to perform, suffered from it severely; while a body of many hundred people, employed in digging and cleaning out the beds of stagnant, brackish, and extremely putrid waters, equally during the heats of the season as during cold and rainy weather, entirely escaped. This immunity is the more remarkable, inasmuch as many of them laboured during the night, for the purpose of preventing the accumulation of water, and were of course exposed, with very scanty clothing, to the utmost vicissitudes of heat and cold, and to all the exhalations and depositions of the very tainted air in which they worked."* These facts are directly opposed to the laws which govern malaria, which is capable of being conveyed to a certain distance by the wind, but never travels against it; which is usually generated in low, filthy, or in marshy situations, and seldom extends far from the place which gave it birth.

At the commencement of the epidemic, much importance was attached by some medical men in India, especially by Mr. Orton, to sol-lunar influence, as an exciting cause of the disease; but subsequent experience has decided against this opinion. The number of cases of cholera in the Madras army, for a period of about two years, amounted to 7664; "and of these it appears that 3725 were admitted into hospital during the quarters of the new and full moon, which, according to Mr. Orton, are the morbific periods; and 3939 were admitted during the first and last quarters, which, according to that author, are non-morbific periods. Thus, between the morbific and non-morbific there is only the difference of 214 cases in 7664, and the excess lies on the side of the non-morbific. We may hence safely conclude that cholera is not directly affected, in individual cases, by sol-lunar influence."†

* Madras Report. † Ibid.

It has been very generally supposed that certain articles of food act as exciting causes of the disease; and several facts have been already mentioned which appear to countenance this opinion. Sporadic cases, it is well known, are produced in all countries by unripe fruit; and it is generally admitted in India that cold rice and curds, and cold drinks, frequently act as exciting causes. In fact, it may be said that any thing which has a tendency to occasion a catarrhal action in the mucous membranes may act as an exciting cause, and ought therefore to be carefully avoided during the prevalence of the epidemic; while warm and tonic substances are likely to be beneficial. Accordingly, the Madras Medical Board, in its instructions for guarding against the disease, recommended a general spicy diet. Bad rice, or what is called ooze rice, in India, has not only been considered to act as an exciting cause, but has been even thought to be the sole cause of the epidemic; and this opinion excited at one time much attention, from the persevering manner in which it was supported by Dr. Tytler, of Bengal, who even went so far as to change the name of the disease to that of *Morbus oryzeus*. This theory has now very few, if any advocates; and it is notorious that all persons, whatever may be the nature of their victuals, are liable to be attacked. Certainly, articles of diet undoubtedly predispose to, or act, as exciting causes of the disease, but there is not a single reason that would lead us to suppose them to have a more extensive influence.

The epidemic has been supposed to be owing to certain changes or peculiarities in the electrical state of the atmosphere; but this theory rests upon such slender grounds, and, from the present imperfect state of our knowledge respecting the constitution of the atmosphere, is so little susceptible of being fully developed, that it affords us little room for discussion. There can be no doubt that the air is often affected by changes which are not indicated by our most delicate instruments, but which are nevertheless very sensibly felt in our frames. Who has not observed the oppression and uneasiness which, to all appearance, is shared by all animals before a thunder-storm, and contrasted these with the feelings of buoyancy and exhilaration which have succeeded when the weather has become serene? Now, it is a curious fact, that the cholera has often been observed in India to have been cut short by a thunder-storm. An instance of this sort has been already noticed in the history of the disease, as having occurred at Madras in 1818; and another fell under my own observation at Kulladghee in 1824, when the epidemic, after having continued for about a fortnight in the troop of horse-artillery stationed at that place, was suddenly cut short by a severe storm of thunder and lightning. We may therefore reasonably expect that the science of meteorology, when brought to a higher state of perfection by extensive and accurate observations, and by the aid of more delicate instruments than we now possess, will detect many properties of which we have at present no conception, and which will afford explanations of numerous diseases, and other phenomena of organic bodies. But until then, while we strive by experiment, and a severely strict mode of reasoning, to add to our knowledge, we must not hesitate to confess our ignorance, and to abstain from conjecture and hypothesis, which might lead us away from the truth, and, by obscuring it, might induce us to relax our efforts in its search.

Not to refer the cause of cholera, then, to any indefinite and unintelligible property of the earth or air, I will attempt to generalize the facts connected with the subject already in our possession, and which have been detailed in the preceding chapters.

1. Cholera may originate spontaneously in places which have been long free from it; as shewn by its repeated occurrence in India, and by its occasional appearance in other countries.

2. Unusual and disturbed states of the atmosphere have generally been found to precede its appearance, and may therefore be considered to be favourable to its development.

3. The concourse of great numbers of people; exposure to the inclemencies of the weather; fatigue, filth, and poverty; draughts of cold fluids; unripe fruit, and saline purgatives, have been always found to act as exciting or predisposing causes of the disease.

4. The epidemic influence is capable of spreading in all directions over great tracts of country, and across a wide expanse of ocean, without being conveyed by individuals in the form of contagion, or wafted in the form of gas or vapour by the air, like malaria. Quarantine observances are therefore incapable of arresting its progress.

5. One attack of the disease does not secure an individual against a second.

6. A clear serene atmosphere, and a great elevation above the level of the sea, have generally been found hostile to the disease.

7. This bears a very striking analogy to other epidemic diseases; all of which have their seat in the mucous system, are considered not to be contagious, affect all situations and every description of people, and one of them (the influenza of 1803,) stretched over a surface equal to that already visited by the cholera; viz. from the confines of China to the western parts of Europe, and thence across the Atlantic to North America.

*Treatment of Cholera.*

The great value of a correct knowledge of the pathology of a disease consists in its making us acquainted with those morbid conditions, the removal of which will restore the body to health. If then the preceding views be correct, we have gained a most important desideratum. We know what those morbid conditions are in cholera against which our remedies must be directed, and the removal of which must form the grand object of our treatment. Accordingly, in the catarrhal cholera, there will always be two principal indications of cure; viz. to remove the diseased action of the mucous membranes, and to restore the circulation of the blood towards the surface. The first will always be present; the second only after the disease has made some progress, and in all severe cases. But, in order to effect these indications, we shall require to employ different means under different circumstances, and to vary our remedies according as certain symptoms predominate, or are wanting. We cannot expect therefore to discover any remedy or specific that will be applicable in all cases; and it is clear that there is just as much necessity for a practitioner to exercise his judgment in treating this, as in treating any other disease in the whole range of the nosology.

*Bloodletting.* It is generally admitted, in India, that bleeding is one of the most useful remedies we possess in the treatment of cholera. I conceive that it ought to be practised not only when there is increased action, but even in every case in which blood can be obtained, however much its quantity may have been diminished towards the surface; except only when there had been great debility previous to the attack. It appears to act as an indirect excitement; it causes the heart and blood-vessels to propel the blood with great energy towards the surface; unloads the congested vessels of the viscera, and thus restores the balance of the circulation. Even in the advanced stage of the disease, when the pulse has been scarcely perceptible at the wrist, I have known it to rise as the blood continued to flow, and the circulation to be restored after the abstraction of twenty or thirty ounces. Numerous cases of a similar description have been published in the Indian Reports. A remarkable effect also which is produced by venesection, is the change in the state of the blood, which, from having been thick and of a dark colour, becomes, after

MANCHESTER

the abstraction of a certain quantity, thinner, and of a natural red. "This is the change, (says Mr. Annesley,) which should always be looked for, and, whether it takes place after the abstraction of one ounce or thirty, is of no consequence; this change must supervene before the patient can be considered safe. Under all circumstances, therefore, I think we should never forego a trial of the lancet." This alteration in the state of the blood is probably to be attributed to the removal of the diseased condition of the mucous membranes.

After the first stage, it is always difficult to procure blood from a vein, on account of its diminished quantity towards the surface, and also of its thickened state, and the slight action of the vessel preventing its ready flow. Our efforts, however, ought to be persevered in as long as there is the least chance of success; if it does not flow readily from one arm, a vein ought to be opened in the other, at the same time, and leeches applied to the temples. The reason why I recommend this situation is, that I always put a blister on the abdomen, and the temples are then the most convenient place for their application.

"Few remedies, on a fair trial, have been more generally and unequivocally advocated than free bloodletting; and the most that has been urged against it is, that it is not always successful."* I have often found it to be most beneficial in the worst description of cases; but it ought not to be depended upon alone, for this is a disease whose rapidity requires the most prompt and decisive measures, and that we should call all our forces into action as quickly as possible; and, while we solicit the circulation towards the surface by means of bloodletting, we ought to urge it on by stimulants given internally, and by blisters and sinapisms applied to the surface.

I conceive that the success of bloodletting depends in a great measure on the quantity abstracted. It has been often observed that, after a small quantity of blood has been withdrawn, the veins of the arm are emptied, no more blood is propelled into them, collapse continues, and the patient sinks. But, on the other hand, when we succeed in keeping up a

* Madras Report. I have been lately favoured with the perusal of a letter from a Russian physician in Moscow to a gentleman in London, in which it is stated that bloodletting had been found there to be more hurtful than salutary; but at the same time the author recommends the application of leeches to the epigastrium. In India, leeches were never had recourse to, except where blood could not be obtained from a vein; and, in the absence of more definite information from Russia, I do not hesitate to give it as my opinion, that venesection, as employed in India, in conjunction with other means, will be found to be a most powerful remedy against the disease; for it would appear, from all that I can learn, that the symptoms of the epidemic in both countries are the same.

steady stream from the vein, and in thus causing a current from the interior towards the surface, we may entertain great hopes of success; and, in order to obtain this happy result, we ought not to allow our efforts to relax until all hope has fled. I have seen cases in which, upon a vein being opened in the arm, the blood has come from it at first only in drops, but, after long perseverance, by means of frictions to the arm and the application of warmth, the blood has at length flowed in a steady stream, has changed its colour, and the circulation towards the surface has been restored.

*Blisters and Sinapisms.* These are among the most beneficial, and certainly the safest remedies, that can be employed in cholera. Their mode of action is quite evident, from what has been said concerning the pathology of the disease. It has long been proverbial in medicine that there is always a determination of blood towards a part which is stimulated. By stimulating the skin, therefore, by epispastics and rubifacients, we restore the circulation of the blood towards the periphery, and thereby relieve the internal vessels, and consequently moderate the diseased action of the secretory apparatus of the mucous membranes.

I generally apply a strong cantharides plaster to the abdomen, and sometimes to the chest; cataplasms of mustard and capsicums to the feet and legs; and hot sand,* or friction, to the arms and hands. It is, I think, only in extreme cases that we ought to have recourse to boiling† water or acid for the purpose of raising a blister;‡ for when there is sufficient time to admit of a blister being raised by means of a plaster, the constant stimulus which it keeps up appears to be more effectual in occasioning a steady determination towards the surface, than the sudden and violent stimulus of the former.§ However, in two or three almost hopeless cases, I have seen the boiling-water blisters attended with the most favourable results. One of these was the case of a native woman in Darwar, who had been ill the greater part of the night. Her friends did not apply to me for medicines till nine o'clock in

* It has been suggested to me by a Russian gentleman, M. Smirnoff, that chaff would answer equally well, and would be pleasanter to the feelings of the patient. Either it, or the sand, may be heated up to 120 Fahr., enveloped in a soft cloth, and thus applied to the parts to be heated.

† The following is the most convenient method of raising a blister by means of boiling water: A cloth, slightly twisted, must be held by its two ends, and its middle part must thus be dipt into a pot of boiling water; when well soaked, it must be suddenly drawn out, and instantly applied to the skin.

‡ I have never succeeded in raising a good blister by means of acid, which has only acted as an escharotic; but the skin may be rubbed gently with it before the application of the cantharides plaster, in order to hasten its effect.

§ Vide Orton on Cholera, p. 418.

the morning. She then had no pulse at her wrist; her features were collapsed, and her skin cold. I ordered the boiling water to be applied immediately to the abdomen, and that she should have a little tincture of capsicums in some warm water. This was done: the circulation returned to the surface; and before the evening she was free from the disease.

*Stimulants.* The most difficult part of the treatment of cholera is the management of general stimulants. Most of them, when judiciously employed, may be productive of benefit; but there can be no doubt that they have sometimes done harm. It is therefore an object of the first importance to ascertain the principles which ought to guide us in the exhibition of them. When applied to a mucous membrane, they stimulate the arterial capillaries, have thus a tendency to produce inflammation, and increase the action of the circulation. It is also probable that they check the secretion of the membrane. Their utility therefore is evidently confined to cases of the pure catarrhal cholera, and they are counter-indicated by pain and burning at the epigastrium, or in the abdomen, which they always aggravate. Alcohol, æther, ammonia, various stimulating tinctures, essential oils, bitters, tonics, and aromatics, have all been used with success in the low form of the disease; and especially when combined with bloodletting, blisters, external heat, and calomel; but all of them ought to be avoided when inflammation is present.

Ammonia, besides its stimulating property, may perhaps have some effect by dissolving the fibrinous matter which lines the inner surface of the bowels, and thus by allowing the other medicines to come in contact with the mucous membranes. The grey stools, which have been already described as a favorable symptom, are probably owing to the combination of ammonia and calomel with the cholera secretion, and appear therefore to point out this medicine as having a favorable effect upon the disease. It is astonishing what large quantities of stimulants may be given with impunity in the catarrhal cholera; but it must be always kept in mind that, as soon as the healthy action of the gastro-enteric mucous membrane is restored, they will begin to produce their usual effects, and will then (if in great quantity) be liable to induce inflammation. Purgatives must therefore be either given along with them, or as soon as the disease begins to take a turn, in order to make them pass rapidly through the bowels, and be discharged by stool. Many of those cases in which great excitement has been known to follow the disease, are, I am convinced, to be attributed to the large quantities of spirits, and other stimulants, which had been given to the patient during the attack, and which might probably have been prevented by a freer use of purgatives. The *drogue amère,* which was much in vogue at one time in India, and was employed in many cases with success, is a compound of stimulants, bitters, and purgatives, and appears well adapted to meet the indications of cure in many cases of the disease.

*Calomel* has been generally recommended in India as a powerful remedy in cholera, but practitioners have not always followed the same indications of cure in its exhibition. Some have given it merely with a view of exciting the biliary secretion; others, under the idea that it has *a specific effect* on the stomach and intestines; some suppose its efficacy to depend entirely on its sialagogue property, and that in this way it cures the disease by a sort of revulsion; its effects, again, have been attributed to its equalizing the circulation, or to its supposed sedative property. I conceive that most of these opinions are more or less erroneous. Its usual action on a mucous membrane, when given in a large dose, is to increase the secretion; in which case it renders the membrane white. If, however, its action be continued long on one spot, it gives rise to inflammation; which has been often observed in cholera; spots of inflammation having been frequently found, after death, in those parts of the mucous membranes to which the calomel adhered. The increased secretion which it produces is generally of a healthy description, is not inordinate nor watery, like that occasioned by the neutral salts, and is accompanied with an increased flow of bile. In regard to its general action on the system, "when given in moderate quantity, it communicates general vigour; it increases the force of the circulation, when this has become languid, by the increased vascular action which it excites; it gives to the blood the disposition to assume the buffy coat; and, by its stimulant operation on secreting organs, it promotes the secretions, and hence acts as a general evacuant."* How does it happen, then, that it has been considered by some, when given in scruple doses, to act as a sedative? This has evidently arisen from its secondary effects only having been taken into consideration, while its appropriate and primary effects have been overlooked.

Now, upon a consideration of the properties which we have just mentioned, we may conclude that, in all probability, calomel would be productive of more harm than benefit in catarrhal cholera, if employed alone; but, when combined with opium or stimulants, there is ample evidence to shew that it

* MURRAY's System of Materia Medica, vol. i. p. 196.

has been highly beneficial. In this form of the disease, our object, in its employment, ought to be to correct the diseased action of the gastro-enteric mucous membrane; to promote the secretion of the glands; to equalize the circulation; and, by its exciting the peristaltic action of the bowels, to cause all the medicines to pass along, and be applied to every part of their inner surface. We are told by Mr. Annesley, that calomel is most useful in removing the fibrinous matter, already described, from the mucous surface of the intestines; which I believe to be the case, and to be another highly important advantage of this medicine. He also supposes that the dark grey stools, whose appearance is always considered to be a most favorable symptom, are owing to the combination of the cholera secretion with the calomel. But I must mention that, by numerous experiments, I found that calomel produces no change upon the pure cholera secretion; and I imagine that the grey colour must be owing to the presence of ammonia, the combination of which with calomel is well known to occasion this colour. It is not improbable, therefore, that the secretion of ammonia by the gastro-enteric mucous membrane is a symptom of returning health. At all events, the occurrence of dark grey coloured evacuations may be always considered as an indication of a favourable change, for they shew that the calomel has done its duty, that part of the viscid fibrinous matter which might have obstructed the action of the other medicines has been removed, and that the peristaltic motion of the bowels has been restored. When bile also is added, a green colour is produced; and, accordingly, evacuations of this colour may be considered as a certain symptom of a return to healthy action in the abdominal viscera.

The usual mode of administering calomel in India has been to place twenty grains upon the tongue, and to wash it down with a draught of warm water and laudanum, or stimulants. The medicines which are given along with it must be regulated by circumstances: when there is no inflammation, stimulants ought to be preferred; when the bowels appear to be torpid, purgatives should be conjoined, or the drogue amère may be substituted. European physicians will probably be startled to hear of the large quantities of calomel which have been employed in this disease, for it was by no means uncommon to give it in repeated scruple doses to the extent of five or six scruples. Small doses in such a disease would be worse than useless; and the want of success in the employment of this medicine in Moscow, may perhaps have arisen from this circumstance not having been attended to.

*Opium* has been much extolled in the treatment of cholera.

Its primary effect is that of a diffusible stimulus; and it diminishes irritability, represses the secretions, and moderates inordinate actions. These properties point it out as a medicine well adapted for the catarrhal cholera; and, when given at the very commencement of an attack, it has often checked the disease. But, beyond this, it ought never to be depended upon alone; and indeed, in the opinion of many able practitioners, its employment, after the first stage of the disease, is considered to be more hurtful than beneficial. In the advanced stages therefore, stimulants may be substituted, or the opium may be employed only in conjunction with calomel. The latter combination was, in fact, at one time much recommended in India, and appears well calculated for fulfilling the intentions of cure in the catarrhal cholera; the requisite properties wanting in the one medicine being supplied by the other. Thus, calomel keeps up a permanent stimulant effect on the system, which opium does not. Opium represses the abundant discharge from the gastro-enteric mucous membrane, while calomel corrects it. Lastly, calomel increases the peristaltic motion of the bowels, and thus effects the discharge of vitiated secretions, while opium relieves irritation. It must at first sight appear paradoxical that purgatives should be required in a disease, one of whose principal symptoms is violent purging, but it has been already stated, in the description of the disease, that purging is sometimes entirely absent; and in many cases, in which there is an almost constant flow of serous fluid from the bowels, their peristaltic motion is nevertheless suppressed, and the fibrinous matter which has been so frequently mentioned is retained. Accordingly, it has been found, in many post-mortem examinations, that the medicines had proceeded no farther than the stomach, and that the intestines were loaded with the cholera secretion. Purgatives, therefore, are indicated in all cases in which the bowels appear not to propel forward their contents, and in which the action of the medicine is prevented by the retention of the viscid fibrinous matter which lines their mucous surface. They are also absolutely necessary in all cases during convalescence, in order to rid the bowels of the stimulating medicines, which would now prove hurtful, together with their diseased secretions; and also to prevent inflammation, which is sometimes a sequela of the disease. From the above observations, it is evident that their employment, during the attack, must be left in a great measure to the judgment of the practitioner. The purgatives which may be employed in the catarrhal cholera, in addition to calomel, are colocynth, jalap, or aloes, combined with some aromatics, or stimulants, or the drogue amère. The neutral salts are clearly inadmissible. Clysters may be used with the same view as purgatives; or stimulating medicines may be thrown into the large intestines, for the purpose of restoring the healthy action of their mucous membrane.

At the commencement of the epidemic in India, the *warm bath* was occasionally employed; but, although the utility of external heat has been universally acknowledged, yet this has been almost always found to be an inconvenient mode of applying it; and in some cases it appears to have been attended with positively injurious effects. Great expectations were at one time entertained of the benefit to be derived from the *vapour bath,* which was first recommended by Mr. Dalton, of the Madras Medical Establishment; but this also, after a fair trial, was abandoned. The same remedy, however, has been revived, with some appearance of success, in Moscow; and the manner of employing it is nearly the same as that recommended by Mr. Dalton in India, as will appear from the following description which has been transmitted to M. Alexander Tourgeneff from a correspondent in Russia. The patient is put in bed, under which are placed two or three basins with burning balls or heated bricks; vinegar, not too strong, is poured upon these, to produce vapours; the bed is covered, and carefully surrounded on all sides, so that the vapour may act upon the whole body of the patient, except his head, without penetrating through the bed-clothes. This is called a vapour bath; and a bed or machine has been already contrived for this operation." Now, as this remedy was not employed in Moscow until the disease had begun to decline, it is not improbable that the success which appeared to attend it was to be attributed to the less degree of malignancy which is known always to characterise those cases which occur when the malady is subsiding.

Experience has led medical men in India invariably to prefer *dry heat;* for which purpose, heated sand, or chaff, as already described, or bottles of warm water wrapped in cloth, may be employed.

It is not necessary to give an account of the various *empirical remedies* which have been recommended. They have either been ascertained to be entirely useless, or to be applicable only to particular cases. In a treatise on any disease, it is of most importance to point out correctly *the principles of treatment;* for it would be almost impossible to enter into details which would be applicable to all its modifications.

*Cold drinks* are clearly counter-indicated in catarrhal cholera; but there appears to be no reason why tepid drinks should be withheld. I would recommend a weak infusion of ginger, or ginger-tea, as it is called; and if this cannot be had, weak brandy-and-water may be substituted. I know that some practitioners have recommended cold drinks; but, if they have ever proved beneficial, this could only have been the case in the inflammatory or mixed form of the disease, for both theory and experience forbid their use in the catarrhal cholera.

It is unnecessary to describe the treatment of the inflammatory cholera, a disease so well known in all countries, that most physicians are familiar with it. I will therefore only remark, in order to contrast it with the catarrhal cholera, that the indications of cure are to remove the inflammation of the mucous membrane of the primæ viæ, to subdue the spasms, and to prevent congestion; the most effectual remedies for which will be free bloodletting, scruple doses of calomel, followed up by castor oil, frictions; and, in severe cases, in which there appears to be a tendency to congestion, blisters. Simple tepid drinks may be given to alleviate thirst, and stimulants are to be avoided.

In mixed cases, viz. in such as have inflammation added to catarrh, and the symptoms of which have been described in a former chapter, the treatment must be modified; and, the greater the symptoms of inflammation, the more guarded must we be in the employment of opium and stimulants. In such cases we must principally depend upon bloodletting, blisters, and calomel; opium being only given at the commencement, to repress the action of the stomach, and enable it to retain the other medicines. It must be remembered, however, that such cases often degenerate into the worst form of the catarrhal cholera, and must then be treated accordingly.

Recovery from cholera is generally rapid; and a proper attention to the state of the bowels is generally all that is required to ensure a speedy restoration to health. Cases do sometimes occur, however, which are followed by inflammation and organic derangement of the mucous membrane of the stomach and intestines, and demand the utmost skill and attention of the physician; but, as they form diseases distinct from cholera, and which are well known in Europe, their treatment does not require a description here. Happily they are not of frequent occurrence, and may be often prevented by a judicious treatment in the first stages of the disease.

It may be useful now to give a short connected account of how a common case ought to be conducted. It must be constantly kept in mind that the disease is so rapid in its progress, that minutes are of more importance in it than hours in most other diseases; and we must therefore bring our different remedies into operation as quickly as possible. Suppose a patient to be brought to us with symptoms of catarrhal cholera, (for instance, with vomiting and purging, a small pulse, cold skin, &c.) a scruple of calomel must be given to him, and must be washed down with fifty or sixty drops of laudanum in a little warm water, or with a dose of one of the stimulating or antispasmodic draughts, the prescriptions of which are given in the next page. Blood must be drawn by venesection, and by a large orifice, until an alteration be produced upon the circulation, indicated by the pulse becoming fuller, by the blood flowing more freely, and regaining its healthy appearance. A strong blister must be applied to the abdomen, and sinapisms to the legs and feet; and the external heat must be kept up by means of heated sand or chaff, in the manner already mentioned. Should the disease continue, or become aggravated, the pulse ceasing at the wrist, and the coldness or collapse increasing, we must then renew the scruple of calomel every hour, and accompany it with doses of the drogue amère, or of some stimulating draught; and our efforts to procure blood from the arm must be persevered in, or leeches applied to the temples. When the circulation begins to be restored towards the surface, the stools to be of a dark grey colour, and the secretion of urine to be renewed, we may consider the disease to have taken a favourable turn; and, when the dejections become green, we may generally consider our patient as safe. If this stage be accompanied by no reaction, all we have to do is to free the bowels from their diseased secretions, and from the stimulating medicines which had been previously given; which may be best effected by colocynth, jalap, or an infusion of senna with tincture of rhubarb. During the whole attack, the patient's thirst ought to be alleviated by a weak infusion of ginger.

Now I do not pretend to say that this treatment is applicable to all the varieties of the complaint. I have merely given it as an example of how one of the most frequent cases ought to be managed; but, under any circumstances, the practitioner who understands the principles upon which the treatment must be founded will experience little difficulty; and I need scarcely repeat, that the greatest difficulty he will probably have to contend with will be *want of time*.

When the attack has been fairly mastered, we have then only to attend to the convalescence of our patient; when it will be necessary to insist upon the use of the most digestible kind of food, and that in small quantity; to forbid cold drinks, and exposure to cold air; and to keep the bowels in a regular state. These measures will seldom fail to restore the patient soon to his usual state of health; and, in fact, it is wonderful in what a very short period of time (a few hours only in some cases,) is sufficient to rescue an individual from the most aggravated form of the disease, and to bring back all the feelings of health, accompanied only with debility.

PRESCRIPTIONS.

I. Mist. Camphor. lb. i.; Tinct. Cardam. ℥ss.; Spirit Ammon. Aromat. ℥i. M. Two ounces for a dose.

This mixture will be found well adapted for the low form of the disease: it is stimulating, and, at the same time, the ammonia having the property of dissolving the fibrinous matter which lines the alimentary canal, will have the effect of removing it, and of thus enabling the other medicines to act.

II. *Antispasmodic Draught.*

℞. Mist. Camphor. ℥ij.; Tinct. Opii, Castor, *aa* ʒss.; Spt. Ammon. Aromat. min. xx. M. *(Madras Report.)*

This ought only to be given in cases accompanied by severe spasms.

III. *Stimulating Tincture.*

℞. Fruct. Capsici. Annui, Zingiberi, *aa* ʒiv.; Alcohol dilut. lb. ij. M. et post sex dies cola.

Half an ounce may be given in warm water, along with the calomel, as directed in the text.

Should none of these medicines be at hand, simple warm brandy and water may be substituted.

*Drogue Amère.*

℞. Aloes Socot. lb. i.; Gum Myrrh, G. Mastich, G. Benzoes, ad. Colomb, *aa* ℥viij.; Croci Anglici, Rad. Gentianæ, *aa* ℥iv.; Eau de vie, lb. xxxvj.; Genèvre, lb. xij. M. To stand forty days, then filter through paper for use.

This medicine was much employed, and often with great success, at Pondicherry and Madras. It may be given in doses of an ounce, as soon as possible, and be repeated at short intervals. It possesses many of the properties which are likely to meet the indications of cure; being stimulant and tonic, and the purgative it contains being likely to excite the peristaltic motion of the bowels, and thus cause the other medicines to pass through, and the cholera secretion to be discharged.

